# Periodontal inflamed surface area in oral cavity associated with febrile neutropenia in patients with hematologic malignancy undergoing chemotherapy

**DOI:** 10.1038/s41598-022-06485-0

**Published:** 2022-02-15

**Authors:** Hiromi Nishi, Kouji Ohta, Yuri Kuramoto, Hideo Shigeishi, Taiji Obayashi, Yukio Yoshioka, Masaru Konishi, Shuichi Munenaga, Hisao Nagoshi, Tetsumi Yoshida, Noriyasu Fukushima, Naoya Kakimoto, Hiroki Ohge, Hidemi Kurihara, Tatsuo Ichinohe, Hiroyuki Kawaguchi

**Affiliations:** 1 Department of General Dentistry, Hiroshima University Hospital, 1-2-3 Kasumi, Minami-Ku, Hiroshima, 734-8553 Japan; 2 Department of Public Oral Health, Program of Oral Health Sciences, Graduate School of Biomedical and Health Sciences, Hiroshima University, Hiroshima, Japan; 3 Department of Clinical Practice and Support, Hiroshima University Hospital, Hiroshima, Japan; 4 Department of Molecular Oral Medicine and Maxillofacial Surgery, Graduate School of Biomedical and Health Sciences, Hiroshima University, Hiroshima, Japan; 5 Department of Oral and Maxillofacial Radiology, Hiroshima University Hospital, Hiroshima, Japan; 6 Department of Hematology and Oncology, Research Institute for Radiation Biology and Medicine, Hiroshima University, Hiroshima, Japan; 7 Department of Oral and Maxillofacial Radiology, Graduate School of Biomedical and Health Sciences, Hiroshima University, Hiroshima, Japan; 8 Department of Infectious Diseases, Hiroshima University Hospital, Hiroshima, Japan; 9 Department of Periodontal Medicine, Graduate School of Biomedical and Health Sciences, Hiroshima University, Hiroshima, Japan

**Keywords:** Medical research, Risk factors

## Abstract

Febrile neutropenia (FN) is an infectious complication that develops during chemotherapy. Although the oral cavity can be an important infection route, it is unknown whether the oral environment is associated with FN. The present study examined the relationship between the oral environment using periodontal inflamed surface area (PISA), a new periodontal disease parameter, and FN in hematologic cancer patients undergoing chemotherapy. In this retrospective cohort study, 157 patients were divided into FN onset during chemotherapy (n = 75) and the FN negative groups (n = 82). The associations of risk factors related to the intraoral environment were assessed. Logistic regression analysis showed that types of blood cancer (odds ratio 1.98; P < 0.01), use of a high-risk regimen (odds ratio 4.44; P < 0.05), prophylaxis treatment with human granulocyte colony-stimulating factor (G-CSF) (odds ratio 4.15; P < 0.01) and PISA (odds ratio 1.02; P < 0.01) were independent factors associated with FN onset. Finally, propensity score matching was performed between two groups; 37 matched pairs were generated. PISA was significantly higher in the FN group than the FN negative group. There was a significant relationship between PISA and FN onset (P = 0.035). The present findings indicate that periodontitis treatment before starting cancer treatment is recommended as supportive care for preventing FN onset during chemotherapy.

## Introduction

Cancer patients undergoing chemotherapy can be affected by various complications, with one of the major adverse effects known to be myelosuppression, resulting in such conditions as neutropenia, infectious disease, and gastrointestinal symptoms including nausea, vomiting, diarrhea, and oral mucositis^1^. Factors related to such adverse effects are roughly divided into therapy-related and patient-related^2^. While age, gender, performance status (PS), and chronic disease are obvious patient-related factors^3^, the oral environment is also thought to be associated with adverse effects^4^. Following the gut, the oral cavity houses the second most diverse and large microbiota including 700 species of bacteria, while the enormous amount of bacteria in dental plaque is equal to that found in stools^5^. Therefore, poor oral condition is considered to be an important risk factor for complications associated with bacterial infection in patients undergoing cancer chemotherapy.

Febrile neutropenia (FN) is a serious complication that can occur during chemotherapy for various types of cancer, especially hematological malignancy. Severe bone marrow suppression along with fever can appear due to the toxicity of cytotoxic chemotherapy agents, which sometimes develops into a severe infection and can lead to death^6^. The most common cause of FN is thought to be a bloodstream infection^7^. However, the positive rate of bacteria in blood cultures of FN patients has been reported to be low (10–25%), thus most cases are treated without identification of the causative bacteria or source of infection^8,9^. Although the intestinal tract and lungs are possible FN infection routes, it is unknown whether the oral environment during cancer treatment, such as periodontal disease, is associated with development of this complication.

The conventional classification of periodontal disease is based on clinical gingival attachment level (CAL), an index used to represent inflammation in the past and present for determining the prognosis of individual teeth^10^. Recently, a new method was reported by Nesse et al. for quantification of the present inflammatory burden posed by periodontal disease, termed periodontal inflamed surface area (PISA)^11^. This evaluation method uses the sum of probing pocket depth of bleeding on probing (BOP)-positive sites for the complete dentition for determining the present inflammatory burden in individuals with periodontal disease^11^. Nesse et al. also showed a dose–response relationship between PISA and HbA1c in patients with type 2 diabetics^12^, suggesting that PISA may be an indicator for examining the relationship between periodontal disease and FN.

We speculated that the oral environment during cancer chemotherapy may be associated with FN. In the present study, the relationships among various factors representing oral status, as well as PISA and FN in hematological cancer patients undergoing chemotherapy were examined in a retrospective manner.

## Results

Two hundred thirty-five patients with hematologic malignancy receiving chemotherapy were initially registered, of whom 78 with poor medical records or inadequate intraoral examinations were excluded from analysis. The baseline characteristics of the 157 enrolled patients are shown in Table 1. Mean age at the start of treatment was 62.3 ± 14.8 years and 81 (51.6%) patients were male. The hematologic malignancy diagnosis was malignant lymphoma in 69, leukemia or myelodysplastic syndrome in 53, multiple myeloma in 34, and chronic active Epstein-Barr virus infection in 1. Onset of FN was noted in 75 (47.8%) at least once during chemotherapy. During the first cycle, the number of patients receiving G-CSF was 76 (49.0%) (Table 1).

**Table 1:** Baseline clinical characteristics of patients in this study.

Clinical parameter	n = 157
Age, years	62.3 ± 14.8
Gender, male/female	81/76
BMI, kg/m^2^	21.6 ± 3.7
**Smoking status, n (%)**	
No	84 (53.5)
Former	49 (31.2)
Current	11 (7.0)
**Alcohol status, n (%)**	
No	79 (50.3)
Occasionally	34 (21.7)
Daily	30 (19.1)
Hypertension, n (%)	52 (33.1)
Diabetes mellitus, n (%)	20 (12.7)
Hyperlipidemia, n (%)	27 (17.2)
Previous episode of malignant tumor	33 (21.2)
Performance status, median (IQR)	0.11 (0.04–0.36)
**Types of blood cancer, n (%)**	
Malignant lymphoma	69 (44.0)
Leukemia/myelodysplastic syndrome	53 (33.8)
Multiple myeloma	34 (21.7)
Others	1 (0.6)
Febrile neutropenia onset, n (%)	75 (47.8)
High-risk regimen, n (%)	131 (83.4)
G-CSF use as primary FN prophylaxis	76 (49.0)

IQR, interquartile range.

**Figure 1:**
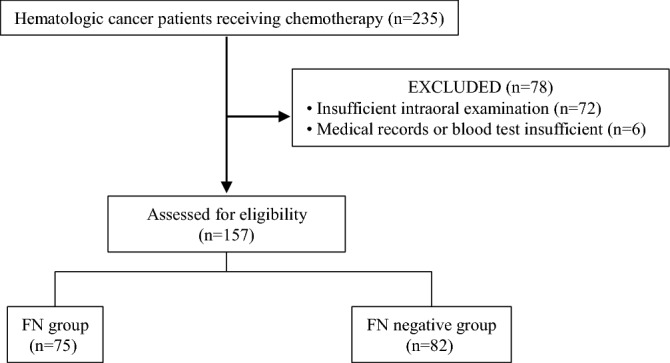
Flowchart showing patient selection.

The 157 enrolled patients were divided into the FN (n = 75) and FN negative (n = 82) groups, based on the standard definition of FN (Fig. 1). When clinical characteristics were compared between the groups (Table 2), there were significantly fewer with malignant lymphomas in the FN group (P < 0.05) (Table 2). On the other hand, leukemia/myelodysplastic syndrome (P < 0.0001) and younger age (P < 0.05) were more common in the FN group.

**Table 2:** Comparison of clinical characteristics between FN and FN negative groups.

Parameter	FN negative (n = 82)	FN (n = 75)	P-value
Age, years	65.1 ± 14.4	59.3 ± 14.6	0.01*
Gender, male/female	38/44	43/32	0.16
BMI, kg/m^2^	21.3 ± 3.7	21.9 ± 3.6	0.29
**Smoking status, n (%)**			0.74
No	47 (61.0)	37 (55.2)	
Former	24 (31.2)	25 (37.3)	
Current	6 (7.8)	5 (7.5)	
**Alcohol status, n (%)**			0.71
No	42 (54.6)	37 (56.1)	
Occasionally	17 (22.1)	17 (25.8)	
Daily	18 (23.4)	12 (18.2)	
Hypertension, n (%)	28 (34.2)	24 (32.0)	0.78
Diabetes mellitus, n (%)	13 (15.9)	7 (9.3)	0.22
Hyperlipidemia, n (%)	12 (14.6)	15 (20.0)	0.37
Previous episode of malignant tumor	18 (22.2)	15 (20.0)	0.73
Performance status, 0–1/2–4	66/15	53/22	0.11
**Types of blood cancer, n (%)**			0.0004*
Malignant lymphoma	44 (53.7)	25 (33.3)	
Leukemia/myelodysplastic syndrome	16 (19.5)	37 (49.3)	
Multiple myeloma	21 (25.6)	13 (17.3)	
Others	1 (1.2)	0 (0.0)	
High-risk regimen, n (%)	59 (72.0)	72 (96.0)	< 0.0001*
G-CSF use as primary FN prophylaxis, n (%)	30 (36.6)	47 (62.7)	0.001*
Remaining teeth	22.7 ± 6.3	23.0 ± 6.9	0.75
**Probing depth, mm, n (%)**			0.95
< 4	21 (25.6)	18 (24.0)	
≥ 4 and < 6	34 (41.5)	33 (44.0)	
≥ 6	27 (32.9)	24 (32.0)	
BOP, n (%)	73 (89.0)	65 (86.7)	0.65
PESA: periodontal epithelial surface area	1006.0 ± 358.7	1107.4 ± 392.3	0.09
PISA: periodontal inflamed surface area	159.7 ± 190.2	338.2 ± 328.3	< 0.0001*

*P < 0.05 (statistically significant).

**Figure 2:**
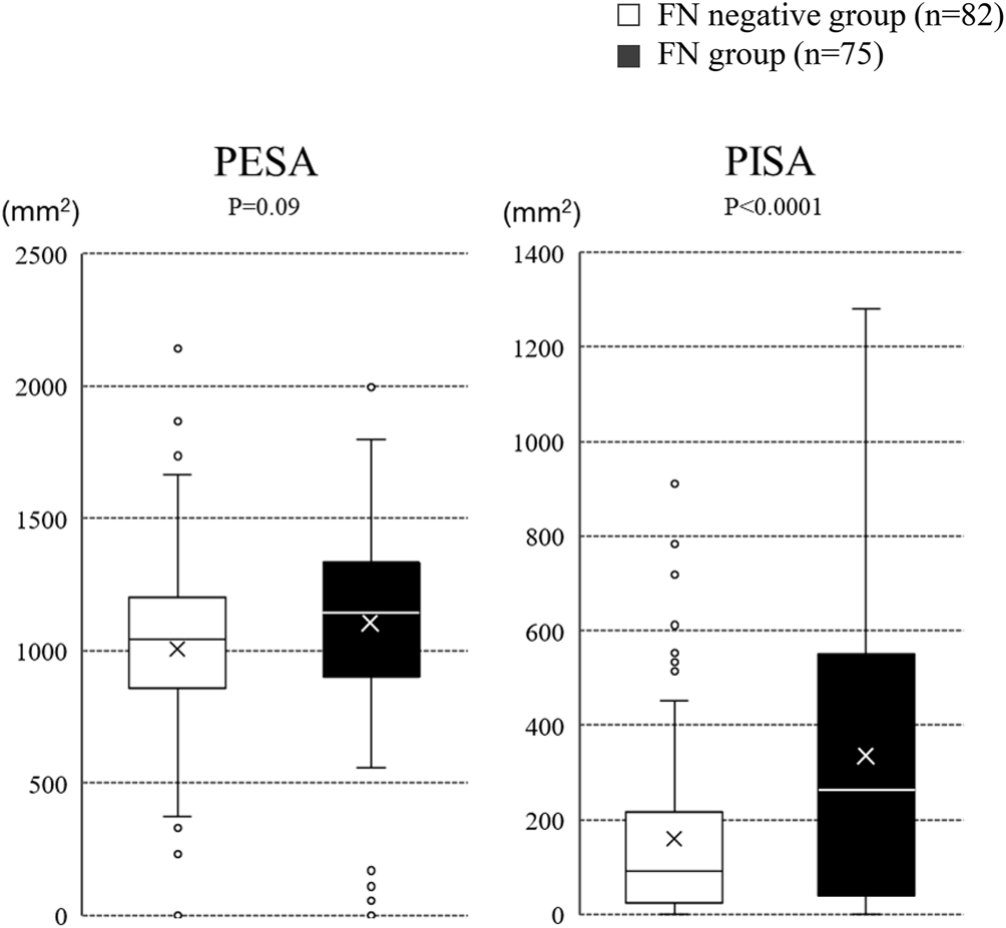
PESA and PISA in FN and the FN negative groups. PESA was not significantly different between the groups, whereas PISA was significantly higher in the FN group (P < 0.0001) (Welch’s t-test).

FN was more often seen in patients treated with chemotherapy regimens known to be associated with high FN incidence, such as anthracyclines, specific alkylating agents (cyclophosphamide, ifosfamide), platinum (cisplatin), etoposide, and cytarabine (P < 0.0001). Additionally, G-CSF use as primary FN prophylaxis was higher in the FN group (P < 0.01) (Table 2). In contrast, older age, body mass index, and history of malignancy were not correlated with FN occurrence. To investigate the oral environment related to FN, periodontal status were analyzed^13^. There were no significant differences between the FN and FN negative groups in regard to remaining teeth, nor for the numbers of patients with periodontal probing depth less than 4, 4–6, or more than 6 mm, or with positive bleeding on probing (Table 2). Periodontal epithelial surface area (PESA) and periodontal inflamed surface area (PISA) are parameters related to periodontal diseases^14^. PESA was not significantly different between the groups, whereas PISA was significantly higher in the FN group (P < 0.0001) (Fig. 2). Variables with a P-value < 0.05 in univariate analysis were subjected to multivariate logistic regression analysis using a forced-entry method (Table 3). P values for the Hosmer–Lemeshow test were 0.66, indicating a good fit of the model. Those results showed that types of blood cancer (odds ratio 1.98, 95% CI 1.25–3.13, P < 0.01), use of a high-risk regimen (odds ratio 4.44, 95% CI 1.12–17.59, P < 0.05) and G-CSF as the primary means of prevention of FN (odds ratio 4.15, 95% CI 1.76–9.80, P < 0.01) were independent factors related to FN, while PISA was also found to be an independent factor associated with FN (odds ratio 1.02, 95% CI 1.01–1.04, P < 0.01).

**Table 3:** Multivariate logistic regression analysis to identify predictive factors for FN.

Risk factor	Odds ratio	95% CI	P-value
Age	0.99	0.96–1.01	0.299
Blood cancer type	1.98	1.25–3.13	0.004*
High-risk regimen	4.44	1.12–17.59	0.034*
G-CSF use as primary prevention of FN	4.15	1.76–9.80	0.001*
PISA: periodontal inflamed surface area	1.02	1.01–1.04	0.004*

*Based on 5 factors included for analysis showing a P value < 0.05 by univariate analysis, conducted using a Mann–Whitney U test, chi-square, or univariate logistic regression test. *P < 0.05 (statistically significant in multivariate logistic regression via forced-entry method).

Finally, propensity score-matching was performed between FN and the FN negative groups using propensity scores generated from 14 clinical factors. In total, 74 propensity score-matched patients (37 patients in matched pairs) were evaluated by univariate analysis. None of the 14 clinical variables were significantly associated with FN (Table 4). Next, PISA was compared between FN and the FN negative groups (Table 5). PISA was significantly higher in the FN group, compared with the FN negative group (P = 0.035).

**Table 4:** Comparison of clinical characteristics between FN and the FN negative groups after propensity score-matching.

Parameter	FN negative (n = 37)	FN (n = 37)	P-value
Age, years	60.3 ± 17.3	60.7 ± 14.7	0.93
Gender, male/female	21/16	23/14	0.64
BMI, kg/m^2^	21.2 ± 4.5	21.8 ± 3.9	0.55
**Smoking status, n (%)**			0.83
No	19 (51.4)	19 (51.4)	
Former	16 (43.2)	17 (45.9)	
Current	2 (5.4)	1 (2.7)	
**Alcohol status, n (%)**			0.95
No	23 (62.2)	22 (59.5)	
Occasionally	8 (21.6)	8 (21.6)	
Daily	6 (16.2)	7 (18.9)	
Hypertension, n (%)	12 (32.4)	15 (40.5)	0.47
Diabetes mellitus, n (%)	2 (5.4)	5 (13.5)	0.23
Hyperlipidemia, n (%)	4 (10.8)	7 (18.9)	0.33
Previous episode of malignant tumor	8 (21.6)	9 (24.3)	0.78
Performance status, 0–1/2–4	29/8	29/8	1.00
**Types of blood cancer, n (%)**			0.95
Malignant lymphoma	14 (37.8)	15 (40.5)	
Leukemia/myelodysplastic syndrome	14 (37.8)	14 (37.8)	
Multiple myeloma	9 (24.3)	8 (21.6)	
Others	0 (0.0)	0 (0.0)	
High-risk regimen, n (%)	32 (86.5)	34 (91.9)	0.45
G-CSF use as primary FN prophylaxis, n (%)	17 (45.9)	17 (45.9)	1.00
Remaining teeth	22.8 ± 7.4	23.0 ± 6.2	0.92
**Probing depth, mm, n (%)**			0.33
< 4	13 (35.1)	8 (21.6)	
≥ 4 to < 6	14 (37.8)	14 (37.8)	
≥ 6	10 (27.0)	15 (40.5)	
BOP, n (%)	30 (48.4)	32 (51.6)	0.53

**Table 5:** Comparison of PESA and PISA between FN and FN negative groups.

Parameter	FN negative (n = 37)	FN (n = 37)	P-value
PESA, periodontal epithelial surface area	1014.9 ± 448.1	1115.2 ± 407.8	0.25
PISA, periodontal inflamed surface area	163.1 ± 235.2	349.5 ± 387.7	0.035*

*P < 0.05 (statistically significant).

## Discussion

This retrospective study was conducted to assess the relationship between risk of FN onset and oral environment in patients undergoing chemotherapy for hematological malignancy. FN is the most common cause of death among complications of chemotherapy and when it causes a medical emergency, prompt treatment of infection is required^6,15^. The etiology of FN at the onset of infection is unknown, though knowledge of causative bacteria is vital as the infection can rapidly progress^16^, with FN management guidelines presented by The Infectious Diseases Society of America available to show appropriate treatment for affected patients^17,18^. In those guidelines, the digestive tract, including the oral cavity, esophagus, colon, and rectum, is considered to be the most common source of infection in neutropenic cases and patients with suspected FN must undergo initial physical assessments related to possible infection^19^. Although the oral cavity is thought to be one of the infection sources in neutropenic cases, the association between the oral environment and FN during chemotherapy remains to be elucidated. The results of the present study show that PISA, which indicates the burden of inflammation caused by periodontitis, is associated with FN. Periodontal disease is comprised of infectious and inflammatory conditions brought about by interactions between gingival bacteria and host inflammatory response^20^. Thus, the oral cavity may be a potential infection source causative of FN in patients undergoing chemotherapy for hematologic malignancy.

Reports regarding risk factors for FN in patients with hematological malignancies who are receiving chemotherapy have been presented. Yokoyama et al*.* examined FN risk factors in patients with non-Hodgkin lymphoma who underwent a regimen comprised of rituximab and CHOP chemotherapy in a retrospective study, and found age, albumin, hemoglobin, and no prophylaxis with daily G-CSF during cycle 1 to be significant^21^. Based on a risk prediction model of FN in patients with hematological cancer undergoing chemotherapy cycles, Moreau et al. found that an aggressive chemotherapy regimen was a significant independent predictor^22^. In the present study, multivariate logistic regression analysis showed that FN onset was associated with PISA, while types of blood cancer, a high-risk regimen and G-CSF use as primary FN prophylaxis were independent factors. Therefore, PISA noted before starting treatment may suggest development of FN during chemotherapy for hematologic malignancy.

Remaining teeth has been shown to be a relevant tool for monitoring periodontal disease status. Some investigators have found a relationship between periodontal disease and risk of cancer. Sakai et al. reported that remaining teeth index values in patients with gastrointestinal cancer were lower in a survey conducted for the Ministry of Health, Labor and Welfare in Japan^23^. The present results indicated that DMF was not significantly different between FN and the FN negative groups. However, Vidal et al. reported that most anaerobic bacteria isolated from anaerobic bloodstream infections in neutropenic cancer patients were oral source of infection^24^. Periodontal bacteria, which are primarily anaerobes may progress to a severe infection such as FN or sepsis during chemotherapy. Therefore, improvement of the oral environment by dental treatment as soon as possible before starting cancer treatment may prevent infectious complications during chemotherapy.

There are numerous reports showing that periodontal disease is associated with onset of systemic disease^25,26^. To evaluate the relationship of the oral environment with periodontal disease, PISA and PESA as periodontal parameter were used in this study. PISA is a newly devised measurement tool used to quantify the amount of inflamed periodontal tissue and found to be useful for assessing the inflammatory burden of periodontitis^27^. Calculation of PISA requires total pocket depth of areas with BOP. It has been reported that BOP can indicate an inflammatory lesion in either epithelium or connective tissue that exhibits a specific histological difference as compared with healthy gingiva^28^. Therefore, PISA was used to distinguish between actively inflamed and non-inflamed or healed periodontal tissue. PISA has been demonstrated to be associated with plaque accumulation and severity of periodontal disease^14^. Additionally, some previous reports have shown a causal relationship between PISA and HbA1c in type 2 diabetic patients^12^, as well as renal dysfunction in elderly Japanese^29^. On the other hand, PESA accurately quantifies the surface area of pocket epithelium when the location of the gingival margin is at or below the cement enamel junction^14^. Therefore, some parts of PESA can consists healthy epithelium that does not contribute to the inflammatory burden. An interesting finding in the present investigation is that the PESA was not associated with FN, whereas PISA was found to have such an association. Thus, assessment of inflamed periodontal pockets, not periodontal pocket depth, is important for predicting occurrence of FN during chemotherapy.

Peripheral neutrophils associated with periodontitis release excess proinflammatory cytokines, such as IL-1β, IL-8, and IL-6, as well as tumor necrosis factor (TNF)-α when stimulated by periodontal pathogens^30^. It has also been shown that proinflammatory cytokines such as TNF-a are increased in serum of patients with periodontitis^31^, while a positive correlation between PISA and increased level of the pro-inflammatory cytokine IL-1a in gingival sulcus fluid has also been reported^23^. On the other hand, pro-inflammatory cytokines including IL-6, IL-8, and TNF were found to be increased in FN patients during febrile periods before and during chemotherapy as compared with healthy controls^32^. Host response periodontal bacteria triggers a pro-inflammatory response and cytokine cascade, which can increase the amounts of inflammatory mediators such as cytokines, and may be involved in FN onset and severity. Conversely, it has been reported that periodontal treatment decreases those proinflammatory cytokines in serum and gingival crevicular fluid^33^. Additionally, Soga et al. reported the case of a 61-year-old male with advanced periodontitis who underwent periodontal treatment in whom FN was reduced during the intervals between chemotherapy cycles, as well as during subsequent courses of chemotherapy and hematopoietic transplantation^34^. Management and appropriate treatment of periodontitis to decrease gingival inflammation prior to cancer treatment may be helpful as supportive care for preventing FN during chemotherapy.

Recent research has shown the efficacy of antibiotic prophylaxis to reduce the incidence of FN in patients during chemotherapy. Several reports have been presented indicating that fluoroquinolone prophylaxis is helpful for patients in whom FN was expected to occur for more than 7 days^35,36^, though resistance to *Escherichia coli* and *Pseudomonas aeruginosa* has been observed in association with that therapy. With the spread of antibiotic-resistant bacteria in medical treatment becoming a severe public health problem^35,37,38^, appropriate use of antimicrobial agents is required. Studies that evaluated fluoroquinolone prophylaxis efficacy have found that prevention of FN in high-risk patients has advantages for infectious complications and does not affect mortality^37,39,40^. In contrast, fluoroquinolone does not reduce the risk for a Gram-positive bacterial infection and what is a suitable antibiotic for prophylactic use remains debatable^41^. Based on these factors, prophylactic antibiotics other than trimethoprim-sulfamethoxazole as prophylaxis against pneumocystis pneumonia are not given to patients undergoing chemotherapy at our hospital. On the other hand, causative bacteria including periodontal bacteria have not been detected in the blood of FN patients and it is unknown whether a periodontal pathogen is associated with this infectious complication. However, oral commensal bacteria, including *Staphylococcus*, the *Streptococcus viridans* group, *S. pyogenes*, and *Klebsiella*, are commonly detected in these patients^42^. Furthermore, pathogenic bacteria, such as *P. aeruginosa*, *E. coli*, *S. pneumoniae*, and *Enterobacter*, are associated with FN and often detected in the oral cavity of patients with poor oral hygiene^43^. Indigenous bacteria, which are mainly composed of Gram-positive bacteria and Gram-negative anaerobic bacteria including periodontal disease bacteria, form a diverse oral microbial flora^44^, which has recently been found to be associated with periodontal health and is known be changed by nonsurgical periodontal treatment^45^. Methods for treatment and management of periodontal disease during chemotherapy that improve the flora associated with periodontal disease possibly associated with FN onset that do not rely on antibiotics should be considered.

This study has some limitations. First, we did not detect specific bacteria causing FN, because pathogens causative of fever in FN patients are often not identified even though blood cultures are examined^46^. In addition, it was conducted at a single center and, though patient care was based on standard chemotherapy guidelines, variations in clinical practice exist and the results may not be generalizable to another care setting. Finally, the results are limited by the use of multiple comparisons. For this study, six factors regarding FN shown to have a P-value < 0.05 in univariate analysis were subjected to multivariate logistic regression analysis. Although it is possible that such analysis can be affected by chance when several variable factors are examined together, we found that FN onset was associated with high PISA, as well as types of blood cancer, with a high-risk regimen for cancer treatment and when G-CSF is used as the primary means of prevention of FN.

In summary, this study is the first to demonstrate that PISA is associated with FN occurrence during chemotherapy in patients with hematologic malignancy. Prior to beginning cancer treatment, management and treatment of periodontitis may be useful as supportive care for preventing FN while undergoing chemotherapy.

## Methods

### Subjects

This enrolled cohort study initially analyzed 235 hematologic cancer patients receiving chemotherapy at Hiroshima University Hospital between July 2017 and March 2020. Approval for this retrospective investigation (number E-453) was granted by the Ethical Committee for Epidemiology of Hiroshima University. Following the provision of a complete description of the study, written informed consent was obtained from all subjects. All methods were performed by relevant guidelines and regulations. The Strengthening the Reporting of Observational Studies in Epidemiology (STROBE) guidelines were used as the checklist for this study^47^. After excluding 78 patients with poor medical records or an inadequate intraoral examination, 157 (81 males, 76 females; mean age 62.3 years, range 20–85 years) with hematological malignancy (malignant lymphoma, leukemia, myelodysplastic syndrome, multiple myeloma, others) and who received chemotherapy were selected for analysis. During chemotherapy, 75 were diagnosed with FN during treatment and the remaining 82 were allocated to the FN negative group (Fig. 1).

FN was diagnosed using the Common Terminology Criteria for Adverse Events (CTCAE), version 4.0^48^. Briefly, those criteria define fever as a single axillary temperature of > 37.5 °C, and neutropenia is defined as a neutrophil count of either < 500/μl or < 1000/μl if the count is expected to decrease to < 500/μl within the next 48 h. Because of the different chemotherapy protocols utilized, those who met the definition of FN at least once during that treatment were considered to have FN. We defined G-CSF prevention as planned use of G-CSF before a neutropenic event during initiation of or during the duration of the first cycle.

All patients received professional teeth cleaning and supragingival scaling within 1 week prior to chemotherapy, as well as instructions related to self-care, including oral hygiene and tongue cleaning with a sponge brush, while tooth and tongue cleaning were performed at least twice a week during chemotherapy. Azulene gargling was prescribed to prevent xerostomia during chemotherapy.

Baseline clinical characteristic data for all enrolled patients, including age, gender, body mass index (BMI), smoking status, alcohol status, hypertension, diabetes mellitus, hyperlipidemia, previous episode of malignant tumor, performance status (PS)^49^, and type of blood cancer, were obtained by retrospective collection from electronic medical records. Results of biochemical tests of blood as well as for FN onset, along with chemotherapy information and G-CSF use were recorded at the end of each chemotherapy cycle. The chemotherapy regimen was selected at the discretion of the treating oncologist using the recommended standard regimen. A high-risk regimen according to ASCO guidelines was defined as that with FN occurrence greater than 20%^19,50^. Hypertension was defined as use of anti-hypertensive medication or confirmed blood pressure of ≥ 140/90 mmHg at rest. Diabetes mellitus was defined as a glycated hemoglobin level ≥ 6.5%, fasting blood glucose level ≥ 126 mg/dl, or use of anti-diabetes medication. Hyperlipidemia was defined as total cholesterol level ≥ 220 mg/dl, low-density lipoprotein cholesterol level ≥ 140 mg/dl, high-density lipoprotein cholesterol level < 40 mg/dl, triglyceride level ≥ 150 mg/dl, or use of anti-hyperlipidemic medication. For smoking status, each patient was categorized as a current smoker, former smoker, or nonsmoker, based on the method of a previous study^13^. Briefly, a current smoker was defined as a patient who had a smoking habit at the time of admission, while a former smoker was defined as a previous smoker who had stopped smoking more than a half year before admission and a nonsmoker as a patient who had never smoked.

### Oral examination

The number of remaining teeth was determined, and periodontal examinations for PISA and PESA were performed as part of oral examination procedures within 1 week before starting chemotherapy. Each enrolled patient also underwent periodontal examinations, including probing pocket depth and BOP. All measurements were performed on fully erupted teeth at six sites per tooth using a Williams periodontal probe (Hufriedy, USA). Bleeding was recorded as either present or absent within 30 s of probing at six sites per tooth. The intra-rater reliability of pocket probing was determined prior to the start of the present study. For that determination, probing depth was assessed at six sites (mesiobuccal, mesiolingual, buccal, lingual, distobuccal, distolingual) in three different subjects two separate times by a dentist. The calculated value of the intraclass correlation coefficient suggesting a reproducible assessment of pocket depth was found to be 0.80. PISA is based on the amount of probing pocket depth of sites with bleeding among the total dentition, whereas PESA is the total amount of surface area of the periodontal pocket. Although CAL is used for calculation of PISA in the original method presented by Nesse, that is difficult to accurately measure as part of a routine clinical examination. In the present study, a previously reported modification of Nesse’s method was employed using periodontal probing depth (PPD) rather than CAL^51^. The values were calculated by use of previously prepared spreadsheets (freely available from http://www.parsprototo.info), as described by Nesse et al.^11^.

### Statistical analysis

Statistical analysis was performed using the statistical software package JMP 12.2 (SAS Institute Inc., Cary, NC, USA). Values are expressed as average, median, and standard error (SE) when an associated variable was a continuous variable, or as frequency and percentage clinically when a discrete variable. The sample size required for use of Mann–Whitney U test was calculated using the G*Power 3.1 software package (Heinrich Heine Universität, Düsseldorf, Germany), with a statistical power of 80%, confidence interval of 0.05, and effect size of 0.5, which showed that a group size of 67 subjects was required. In addition, post hoc power analysis was performed using G*Power with a confidence interval of 0.05 and effect size of 0.5, and the sample size achieved a statistical power of 0.86. The effects of various clinical parameters on FN were evaluated using univariate analysis and independent influences were assessed using multivariate logistic regression analysis. Covariates with P < 0.05 in univariate analysis were entered into multivariate analysis with a forced-entry method. The Hosmer–Lemeshow test result was not statistically significant, suggesting that the model exhibited good fitness. A propensity score-matched analysis was performed to eliminate the effects of clinical confounding factors. Propensity scores were calculated by logistic regression analysis of 14 clinical parameters (i.e., age, sex, BMI, smoking status, alcohol status, hypertension, diabetes, hyperlipidemia, performance status, previous episode of malignant tumor, blood cancer type, high-risk regimen, G-CSF use as primary FN prophylaxis, missing teeth). Caliper of 0.25 standard deviations of the propensity score was used for analysis. All analyses were two-sided tests and a P value < 0.05 was considered to indicate statistical significance.
